# Confronted with COVID-19: Migrant live-in care during the pandemic

**DOI:** 10.1177/14680181211008340

**Published:** 2021-04-09

**Authors:** Michael Leiblfinger, Veronika Prieler, Mădălina Rogoz, Martina Sekulová

**Affiliations:** Johannes Kepler University Linz, Austria; International Centre for Migration Policy Development, Austria; Slovak Academy of Sciences, Slovakia

**Keywords:** Critical workers, cross-border care circulation, long-term care, migrant live-in care, pandemic policies, pandemic response, transnational care arrangements

## Abstract

In the spring 2020, measures introduced across Europe to limit the spread of COVID-19 included, among others, the temporary closure of borders. For Romanian and Slovakian live-in carers, this meant they were no longer able to commute between the Austrian households they work in and their respective countries of origin. Due to the relatively short cyclical rotas of 2–4 weeks, travel restrictions heavily affected cross-border live-in care between the three countries, which makes them a particular case for studying the effects of pandemic-related measures on transnational care arrangements. Drawing on media reports, relevant laws and policies, and interviews with representatives of care workers’ interests, the article examines how live-in care as a whole and care workers in particular were affected by the pandemic and related policy responses such as specific travel arrangements and financial incentives for workers. It shows that while live-in carers were deemed critical workers and essential for the long-term care system, the inequalities and dependencies already existing in transnational care arrangements were deepened. Care workers’ wants, needs and interests were subordinated to the interests of care recipients, agencies and sending and receiving countries.

## Introduction

The employment of live-in care workers has become an important pillar of long-term care systems, especially in familialistic care regimes across Europe. Mainly female carers, typically from Central and Eastern Europe, at least partially fill the care gaps emerging as a result of population ageing, changes in family structures and the increasing labour market participation of women (Casanova et al., 2020; Chau, 2020; Horn et al., 2019; Lutz, 2018; Schwiter and Steiner, 2020). The COVID-19 pandemic drew attention to the importance of (migrant) care work for societies facing enormous pressure on long-term care in general and live-in care in particular. Previously undervalued workers suddenly became critical and their work and services essential. While the older population is most vulnerable to the virus and those who provide care are indispensable, border closures and other pandemic-related measures put a (temporary) halt to the transnational circulation of care workers. Austria, Romania and Slovakia represent a particular case for studying the effects of COVID-19 responses: Austria is a country with a legalised and highly institutionalised live-in care model with almost all live-in carers being self-employed. Roughly 80% of care workers come from Romania and Slovakia and commute to Austria in short-term cyclical rotas typically ranging from 2 to 4 weeks. Due to the relatively short time spans, travel restrictions affected these transnational care arrangements earlier and more intensely than in countries where live-in care workers stay for longer periods.

The article examines how live-in care and its workers were affected by the pandemic and related policy responses; investigates asymmetric power relations, decisions and control over individual actors; and shows how carers’ interests were treated as secondary to the interests of care recipients, agencies and sending and receiving countries. In contrast to the national focus that implicitly denotes live-in care workers as foreigners and ‘mobilises imaginaries of relatively discreet and/or disconnected places of origin and destination’ (Pelzelmayer, 2016: 1707), we do not consider Austria, Romania and Slovakia separately, but rather investigate the interconnected care mobility between Austria on one side and Romania or Slovakia on the other by taking into account the supranational, transnational and national dynamics, regulations, power relations and inequalities the live-in arrangements are embedded in. The position of care workers in the receiving societies, that is, their rights, duties, opportunities and challenges, underlines their legal status (and relevant policies in place).

This article draws on data from media reports, governmental websites and documents, relevant laws, ordinances and official guidelines, and empirically covers the first phase of the pandemic and subsequent responses between March and September 2020. Three interviews were conducted during that period to gain insights into the perspective of Austrian-based stakeholders representing care workers: *CuraFAIR*,^1^ an advice centre for live-in care workers; *DREPT pentru îngrijire* (‘right for care’),^2^ a grassroots organisation of predominantly Romanian live-ins; and the *Institut für Personenbetreuung* (‘Institute for Personal Care’),^3^ a carers’ organisation founded in 2015 by a former live-in care worker from Slovakia. Representatives of these organisations were interviewed to include and assess their perspectives on the situation of live-in carers and the difficulties they faced during the pandemic.^4^ An additional interview was conducted in April 2020 with an agency from Slovakia that brokers care workers to Austria.

After a theoretical outline of intersecting inequalities that mark live-in care, the Austrian model is described. The following section presents the pandemic-related responses in the three countries that affected live-in care, followed by a critical discussion of these measures in the subsequent section. The conclusion shows that during the first phase of the pandemic, care workers’ wants, needs and interests were subordinated to those of care recipients, agencies and sending and receiving countries.

## Intersecting inequalities and multiple vulnerabilities of live-in care workers

Live-in care reflects the ongoing transnationalisation of care that involves the movements and practices of care labour, care capital, care commitments and political actors (Anderson and Shutes, 2014; Williams, 2011). Intersecting structural inequalities crosscut care work at different levels: the micro scale of everyday experiences, where relations of gender, class, race, migrant status, age or sexuality intersect; the meso scale of political, social and institutional factors manifested in national migration, employment and care regimes; and the macro scale of interlinking global capitalism, geopolitical, gendered and post-colonial hierarchies and inequalities (Williams, 2018). Increased economic participation of women in Western European countries has neither resulted in a more equal (re)distribution of care responsibilities within families nor challenged the gendered distribution of care work; it is therefore described as ‘distorted emancipation’ (Uhde, 2016). Gaps have been filled through outsourcing care work to women from less economically developed countries (Hochschild, 2001; Kofman and Raghuram, 2015). As the concept of the new international division of reproductive labour (Parreñas, 2015) explains, (middle and upper class) women in the West use their privilege of race and class to transfer their reproductive labour responsibilities to less privileged women through cross-border care, thereby exploiting class and regional inequalities.

Care workers typically come from regions with fewer employment opportunities, worse labour conditions and/or low wages. The majority of live-in carers are women in the mid and later stages of their working life: in Austria, around 95% are women and three in four over 45 years of age.^5^ Risk of age and gender discrimination constitute additional factors that motivate migration. Based on the example of Slovakian live-in care workers in Austria, previous research underscored the relevance of carers’ low economic status in their country of origin for their position in the receiving country, as carers with experiences of unemployment or poor working conditions are more willing to accept precarious conditions in Austria (Bahna, 2014). For many Eastern and Central European care workers, working in Western Europe remains appealing even when jobs entail poor and exploitative working conditions (Österle and Bauer, 2016; Sekulová and Rogoz, 2019).

Migrant carers often work, regardless of the regulatory system pertaining to live-in carers, in a legal grey area and are usually paid low wages, hidden in the private space of households, where official work, health and safety regulations do not apply, expected to perform demanding tasks, while facing the risk of exploitation and lacking decent labour conditions (Aulenbacher et al., 2020; Lutz, 2018; Winkelmann et al., 2015). The existing literature demonstrates extensively that the private household as a workplace blurs the borders between work and the private sphere of workers. Moreover, the expected emotional dedication of carers and the narrative of being a family member distorts the contractual nature of the relationship of carers and employers (Cox, 2006; Weicht, 2015), which is often based on inequality and power hierarchies (Anderson, 2000). The COVID-19 pandemic highlighted those transnational inequalities and dependencies. While Austria, Slovakia and Romania – like other European countries – closed their borders to prevent an uncontrolled spread of COVID-19, receiving countries such as Austria at the same time stipulated exemptions to guarantee the cross-border mobility of care workers essential to their long-term care systems.

## Cross-border live-in care in Austria

After almost two decades of growth as an irregular, but socially and politically generally accepted mode of long-term care, Austria regulated its so-called 24-hour care model in 2007. The legalisation brought about a comparatively extensive regulatory framework and the recognition of personal care as a profession. This formalised, professionalised and therefore legitimised live-in care and turned the erstwhile informal care workers into recognised self-employed workers. Carers’ legally defined tasks range from housework to medical care, although the latter has to be delegated to them by qualified health care professionals. Given their status as self-employed, regulated working hours, collectively bargained minimum wages, entitlement to paid holidays or weekend surcharges do not apply to live-in carers. Working conditions often show a high degree of precarity and usually include low income, availability (almost) around the clock with little opportunity for breaks and rest, physical and psychological stress and constrained opportunities to be with their own family and friends. Economic reasons are typically the main driving forces to work under these conditions regardless (Bahna and Sekulová, 2019; Melegh et al., 2018; Sekulová, 2013; Weicht and Österle, 2016).

Like in other countries, the Austrian model of personal care is based on workers who are recruited abroad and live in the households of care recipients during their rotas. Typically, two carers alternate and commute between their countries of origin and the place of work. Unlike other countries such as Germany or Switzerland, where stays of 2 or 3 months are not uncommon, care workers’ rotas in Austria typically last between 2 and 4 weeks, as many carers initially came from regions bordering Austria in, for example, the Czech Republic and Slovakia. This arrangement was consolidated by the introduction of an allowance intended to compensate households for the higher costs, as personal carers are liable to pay Austrian social insurance contributions^6^ since their legalisation in 2007: in order for households to receive the full allowance of 550 euros per month, two live-ins have to alternate within the same month^7^ (Österle and Bauer, 2016; Steiner et al., 2019).

Since its legalisation, live-in care has become an important pillar of the Austrian long-term care system. While 25,000 care workers were registered at the end of 2010 (Österle, 2016: 254), their number grew to 62,000 by the end of 2019 (WKO, Economic Chamber Austria, Statistics Department, 2020: 11). The most important countries of origin are Romania, at 47%, and Slovakia, at 33% (as of the end of 2019^8^). Almost 33,000 households^9^ relied on this care model in early 2020 (BMSGPK, Federal Ministry for Social Affairs, Health, Care and Consumer Protection, 2020b). Likewise, the number of agencies that organise personal care rose from 133 at the end of 2011 (Österle et al., 2013: 165) to 826 by the end of 2019 (WKO, Economic Chamber Austria, Statistics Department, 2020: 11). Along with their number and influence through the Economic Chamber, which has mandatory membership, the roles of agencies also increased: apart from recruiting and placing care workers, additional services like the collection of payments or the organisation of travel are usually offered (Leiber et al., 2020; Österle and Bauer, 2016; Schmidt et al., 2016). Due to information and power imbalances between agencies and carers, the latter generally do not have the liberties that typically accompany self-employment (Aulenbacher et al., 2021; Sekulová and Rogoz, 2019). As self-employed and just like agencies, personal carers are obliged to become members of the Economic Chamber, which (formally) represents their interests. In practice, however, self-organised initiatives such as *DREPT pentru îngrijire* and the *Institut für Personenbetreuung* or the trade union organisation *vidaflex* increasingly play a far more significant role (Aulenbacher et al., 2020; Haidinger, 2016).

## Pandemic-related responses in Austria, Romania and Slovakia

### Travel restrictions and measures to re-enable care workers’ mobility

In the first months of the pandemic, travel restrictions and quarantine regulations severely affected care mobility: as a result of measures to contain the pandemic, many care workers, both those who were supposed to commute from Austria to Romania or Slovakia at the end of their rotas and those who were supposed to commute to Austria to replace them, were unable to travel internationally or decided not to, either because of closed borders, quarantine and testing requirements, fear of contagion, job loss, their (changed) situation at home, or because agency-organised mini-buses, which normally play a significant role in care workers’ transnational movements, were not in operation (Koštialiková, 2020; Leiblfinger et al., 2020; Matzal, 2020; Meseșan, 2020; Vojenčák, 2020).

In Slovakia, care workers returning from abroad in April 2020, through state-organised repatriation transport or individually arranged, had to quarantine in governmental facilities where rooms or bathrooms were usually shared. After a negative COVID-19 test result, they were allowed to leave and subsequently had to spend another 14 days in self-isolation at home. Care workers criticised the quarantine facilities because shared transports and rooms exposed them to a higher risk of contagion than home quarantine (Koštialiková, 2020). Although returnees with a negative test result were exempted from obligatory quarantine, Slovakia did not accept tests conducted abroad (including Austrian tests) in April 2020. Beginning in May, those working in the Austrian provinces Burgenland, Lower Austria and Vienna were exempted from obligatory quarantine in government facilities with proof of a negative test result no older than 96 hours (including tests from abroad). However, testing also led to problems: while some care workers had the associated costs covered by the client they cared for,^10^ others had to pay for it themselves.

A limited number of Slovakian care workers reacted to the travel impediments by finding alternative ways of crossing the borders: these transnational movements, often supported by agencies, typically consisted of personally arranged travel to the borders, crossing them on foot and subsequent agency-organised travel to clients’ households (Sekulová, 2020). These border crossings were motivated and justified by a mix of factors: financial hardship and dependence of carers’ families on income from Austria (Bahna and Sekulová, 2019), moral commitment or pressure from agencies and households to return.

Care workers from Slovakia and both Slovakian and Romanian agencies criticised the ban on international travel and requested the re-launch of international taxis which take care workers directly from client households to their homes. This option was considered safer compared to alternatives such as trains or buses (Koštialiková, 2020). The argument put forward was that, during lockdown, carers were in social isolation with their clients for the period of their stays in Austria. International mini-buses observing strict hygienic measures including obligatory face masks for drivers and passengers would further reduce the risk of contagion. However, the objection was raised that the rationale behind the demand for international taxis was purely economic. Instead of supporting care workers, taxi services would serve as a means of profit for agencies, especially if imposed on live-in carers, as is not uncommon for Romanian care workers. International taxi travel from and to Slovakia and Romania was re-launched after the opening of borders in June 2020.

On the Austrian side, efforts were undertaken to (re-)enable care workers to cross borders, for example, through exceptions to travel restrictions by creating transit or care corridors, a demand that had already been raised by Austrian and Romanian agencies during the first weeks of travel restrictions. As live-in carers were not able to cross through Hungary, which closed its border, in the spring 2020, three chartered flights from Romania, Bulgaria and Croatia as well as six special night trains from Western Romania were organised in April and May 2020 (Leiblfinger et al., 2020; Lupu, 2020). With borders reopened, the trains were suspended – also because they were underused. Moreover, agencies were demanding that they and care workers should be allowed their own choice, that is, to use the transport options organised by agencies themselves.

Although no more than 355 live-in carers were flown in and only up to 2000 care workers were able to travel by train to Austria and return to Romania, the initial two flights and the first train received considerable media attention. In Austria, reports depicted three actors as ‘saviours’ of the endangered live-in model: the provinces of Burgenland and Lower Austria that covered the costs of the flights, the respective regional Economic Chambers that paid for the hotel the care workers had to spend their 2-week unpaid quarantine^11^ in after the flights and the brokering agencies involved in organising the flights (Leiblfinger and Prieler, 2020). When using the trains, carers no longer had to remain in 2-week quarantine but were tested on arrival and had to stay in a hotel for one night until the test results were available. However, there was uncertainty about the costs for having to stay in quarantine in case of a positive test, as there was no regulation on who had to pay for the hotel. According to one interviewee, this was apparently a matter of negotiation between individual care workers and their respective agencies. A 2-week hotel bill may correspond to the monthly income of a live-in carer and is therefore not affordable for them.

With regard to the special trains, on the Romanian side, the Ministries for Transport, Infrastructure and Communications, for Internal Affairs, for Labour and Social Protection and for Foreign Affairs were involved in the decision regarding the train corridor (Ministerul Transporturilor, Infrastructurii și Comunicațiilor, 2020). To carry out the agreement, the National Railway Transport Company, local authorities, the Romanian Road Authority and various territorial structures were engaged (CFR Călători, 2020). In Austria, apart from the Economic Chamber, the Federal Ministries for Mobility and for the European Union, and the publicly owned Austrian Railways were involved in the organisation of the trains. These multiple actors and their collaboration were necessary to realise these measures and highlight the importance that state(-related) actors attach to transnational labour migration and, in particular, care mobility.

Given the total number of nearly 30,000 Romanian personal carers working in Austria, however, these measures were symbolic at best (Leiblfinger et al., 2020). According to one interviewee, care workers were poorly informed by their Romanian agencies regarding mandatory quarantine in a hotel or working conditions in Austria. This applied, for instance, to live-in carers who were assigned to new households and not fully informed about the specific care needs of their yet unknown clients. In addition, care workers were at an increased risk of contagion on their journeys and during quarantine periods as, for example, safety measures were rarely observed in (overcrowded) mini-buses (Matei, 2020). Carers interviewed by Romanian media also reported different conditions in the two countries: while the transportation from their homes in Romania to the airport as well as the situation at the airport were described as crowded and with no possibility to maintain physical distance to others,^12^ in Austria, care workers observed physical distancing regulations until they were brought to quarantine facilities. There, two or three carers shared a room and, in fact, were able to meet in larger groups. Moreover, during quarantine, care workers’ documents were taken from them by order of local authorities – only to be returned after a public outcry in Austria (Miedl, 2020). In addition, while care workers remained in quarantine for 2 weeks, the older adult they cared for and their families were not required to do so, nor were they required to be tested (Meseșan, 2020).

### Extended rotas and support measures

Because of (temporarily) closed borders and travel restrictions as well as the higher risk of the older population to become seriously, even fatally ill from COVID-19, safeguarding the live-in care provision received increased media and political attention in Austria, especially in the early phase of the pandemic. In March 2020, the Austrian government announced that it would provide 100 million euros to the social care sector, including family and live-in care. Furthermore, therapy and rehabilitation facilities that had been closed in response to the pandemic were to be adapted for care recipients whose home-based care, be it personal care or family carers, became unavailable. Another proposition was that ‘Zivildiener’, that is, conscientious objectors to compulsory military service who serve an alternative community service, or those in caring and nursing training help fill the anticipated care gaps (Leichsenring et al., 2020a). In practice, these propositions, which indicate the pressure political actors faced and their willingness to propose far-reaching measures, were never enacted on a large scale. Instead, another response proved much more important: many carers worked extended rotas, in some cases up to 3 months, either due to lacking possibilities to travel home, out of a sense of duty towards the cared for and their relatives, but also because agencies and households urged them to show responsibility towards their clients and agencies in a time of crisis (Leiblfinger et al., 2020; see also Giordano, 2021).

As an incentive, Austria introduced a one-time, tax-free bonus of 500 euros^13^ for rota extensions of at least 4 weeks. However, official information was available in German language only – apart from Vienna, where the form to apply for the bonus was also available in Romanian and Slovak. Moreover, application and payment modalities varied between the nine Austrian provinces who were responsible for the pay-out: in Lower Austria, where nearly one in three personal carers are registered,^14^ care recipients had to apply for the bonus and pass it on to the live-ins. Similarly, in the provinces of Burgenland, Carinthia and Styria, the bonus was paid out to the care recipients, while in Salzburg, Upper Austria and Vienna, the bonus went directly to carers. In Tyrol, the bonus was paid out to live-ins directly if they provided an Austrian bank account, which few personal carers were able to as they do not have one, in which case it was also transferred to the care recipients. The province of Vorarlberg had predefined a number of agencies that could apply for their brokered carers in bulk, with those live-ins requesting the bonus without agency help having to apply through the municipality their client lived in. These varying regulations that partly relied on the full cooperation of care recipients made access to the bonus difficult and increased care workers’ dependence on agencies and households. According to one interviewee, some carers deemed the bonus amount ‘laughable’, considering that rotas were extended for up to 3 months. While access to the bonus was very difficult for many care workers, households’ access to the public allowance for 24-hour care was simplified without the need to act: for the duration of the pandemic, it was no longer required to have two care workers alternate in the same month. The financial interests of live-in care recipients evidently outweighed those of the migrants providing care.

As many households reduced family and other visits for fear of contagion (Matei, 2020; see also Horn and Schweppe, 2020), which in some cases provide care workers with an opportunity to rest, extended rotas generally meant increased workloads, longer working hours and isolation for care workers, the latter already an issue prior to the pandemic (Bauer and Österle, 2013; see also Giordano, 2021). In addition, live-in carers carried the mental burden of uncertainty and an indefinite separation from their own homes, families and friends (Leiblfinger et al., 2020; see also Safuta and Noack, 2020; Schilliger et al., 2020). In sum, extended rotas for some care workers led to physical and mental strain. Services like psychological counselling, which the Vienna Economic Chamber offers to live-ins registered in the province, seem like a mere drop in the ocean, especially as there were not enough counsellors who spoke Bulgarian, Hungarian, Polish or Slovak (Leichsenring et al., 2020b).

### Financial measures to support live-in carers

For many live-in carers who could not or did not want to return to Austria, due to travel restrictions, extended rotas by the care workers they normally would have replaced, fear of contagion on the journey, or changing personal situations at home, the pandemic likely resulted in financial hardship. The same applies with regard to the periods of quarantine, since self-employed personal carers are only paid for the days they provide care to their clients. Austria implemented a hardship fund to mitigate negative economic effects of the pandemic on small businesses. As entrepreneurs, personal carers were (and are) eligible for monthly support of up to 2500 euros for a period of initially 3, later 6 months (BMF, Federal Ministry of Finance, 2020). In practice, however, many live-in carers did not have access to the fund because they lacked the required Austrian bank account, tax number or income tax assessment notice. As it was available only in German language, some may also have struggled to fill out the detailed application form. As one interviewee pointed out, there may have been another deterrent: if care workers applied for a tax number as the federal government encouraged them to do, there was uncertainty as to whether an annual income tax assessment would have to be completed in the future – something personal carers typically do not have to do as their income is usually below the tax threshold.^15^ Care workers have also been at a disadvantage concerning another source of financial support: the special, pandemic-related children’s bonus, which all recipients of the family allowance and thus a number of live-ins received in September 2020, was, like the family allowance itself, adjusted to the cost of living in the children’s country of residence (Matei, 2020).^16^

### Lobbying of care workers, brokering agencies and their interest groups

Care workers and brokering agencies shape the way live-in care is organised on the ground and (try to) influence its regulations. This was also visible in the spring and summer 2020. Although live-in carers represent a substantial share of Slovakians working abroad, care mobility received little political attention in Slovakia. To raise awareness of the effects of the imposed measures on care workers and make their voices heard, live-in carers and their representatives organised two protests at the Slovakian–Austrian border. During the first protest in late April 2020, care workers requested exemption from mandatory quarantine after proving a negative COVID-19 test for those working in all federal states of Austria (Bratislavske Noviny, 2020). The second protest in early May 2020 called for the abolition of the obligatory 14-day self-isolation upon arrival in Slovakia, re-opening travel for cross-border commuters, and the re-launch of international taxi services. The government acknowledged the requests, assuring it would ease measures as soon as the pandemic situation in Austria improved (Petrovič and Debnár, 2020).

The general lack of support during the first months of the pandemic offered to transnational care workers by Romanian and Slovakian authorities, respectively, subscribes to these sending countries’ positions with regard to care work migration. Previous research on the perceived impacts of care work migration on the health care, long-term care and education systems revealed that neither Romania nor Slovakia have taken specific steps towards incorporating care work migration into their policies. National level stakeholders consider care migration to have a positive impact on families, sending communities and the countries themselves since care workers’ families benefit from remittances and the opportunity to work abroad translates into lower unemployment in the sending countries (Sekulová and Rogoz, 2019).

Without governmental support, care workers themselves and their organisations launched efforts towards self-support, for example, by sharing information on pandemic-related developments, travel, quarantine and testing regulations, as well as on financial support measures such as the bonus or hardship fund. This exchange took place primarily via social media (see also Safuta and Noack, 2020) and was a direct result of the lack of information from governments, the Economic Chambers which personal carers are members of and agencies, although differences especially by the latter have been pointed out by our interviewees. Despite these developments, organisation on the side of live-ins is rather weak. With regard to Romanian care workers, reports published in the media in March 2020 revealed that they felt abandoned by the Romanian and Austrian authorities alike (Matzal, 2020). In response, from early March onwards, *DREPT pentru îngrijire* was (and continues to be) vocal in highlighting the lack of support that Romanian care workers received in Austria, particularly with regard to initial financial and psychological support that would have helped care workers already in Austria continue their work.

Furthermore, some agencies were actively involved in lobbying efforts to amend imposed measures, defending the interests of carers, but also their own. In Slovakia, a number of agencies started communicating with the institutions in charge of pandemic containment measures, for example, by sending requests to the respective ministries. Similarly, some agencies played an active role in informing care workers about developments concerning the epidemiologic situation as well as the measures, policies and welfare responses that were implemented. Agencies also supported care workers, for example, in applying for the bonus for extended rotas and the hardship fund – for which some agencies reportedly charged up to 20% of the benefit or, particularly in Romania, kept charging monthly commissions (payable for each rota).

## Implications of the pandemic for live-in care

Between spring and late summer 2020, the COVID-19 pandemic underlined the fragility of the Austrian live-in care model. Despite the plaudits for (live-in) care workers, their critical and essential services and their media portrayal as dedicated heroines (Leiblfinger and Prieler, 2020; Van Hooren, 2020), it highlighted that care workers’ interests are subordinated to the interests of care recipients, agencies and sending and receiving countries. The pandemic exacerbated the already precarious working conditions of live-in carers, for example, limited free time and tasks or workload beyond the care workers’ competencies (Österle and Bauer, 2016; Winkelmann et al., 2015). This has reproduced the nationality- and gender-related inequalities the live-in model is based on (Leiblfinger et al., 2020; see also Lutz, 2020; Schilliger et al., 2020).

The pandemic once again highlighted care workers’ unequal relationship with agencies which manifests through an imbalance of information and power of negotiation with regard to their working arrangements. According to one interviewee, personal carers are yet to learn to negotiate with both agencies and care recipients or their families, including their remuneration, work schedule, means of transportation, as well as the commissions imposed by agencies.^17^ An already physically and psychologically demanding work was pushed towards exhaustion through the extension of care workers’ rotas from typically 2–4 weeks to up to 3 months during the first phase of the pandemic. Moreover, self-employed care workers in Austria did (and do) not have the same access to health and social protection like paid sick leave as those working on the basis of standard employment, while psychological support was (and is) available to live-in care workers only to a limited extent.

The measures implemented in response to the pandemic-related restrictions revealed and to a certain extent exacerbated the already existing power imbalances and dependencies. Applying for support from the hardship fund was possible in technical German language only, which, according to our interviewees, made it difficult for care workers to obtain the aid. The varying regulations that partly relied on the full cooperation of care recipients made access to financial support difficult and increased care workers’ dependence on households and agencies. This demonstrates not only power imbalances within the live-in care model but also the pivotal role agencies play in live-in care (Leiblfinger et al., 2020; see also Habel and Tschenker, 2020), which was prevalent even prior to the pandemic. Moreover, international travel restrictions introduced immediately after lockdown required care workers to present negative COVID-19 test results or remain in unpaid quarantine before starting their rotas. At least in the first months of the pandemic, care workers were expected to pay for the tests themselves, indicating that the (financial) interest of care recipients outweighed those of live-in care workers. Whether changes in regulations resulted from agencies’ and/or care workers’ lobbying efforts or were primarily the result of a general improvement of the pandemic situation remains an open question. However, due to lobbying efforts, governments and general crisis staff were made aware of specific problems that live-in care workers were confronted with as cross-border commuters. As the pandemic situation became less dramatic, measures were suspended without hesitation.

Furthermore, personal carers already in Austria were urged by agencies (and households) to show responsibility towards their clients in a time of crisis. Therefore, agencies were reproducing the narrative of care workers’ moral obligation and responsibility and invoked an imperative of mutual solidarity that put further pressure on live-ins. This circumstance – against the backdrop of the difficulty in accessing the bonus for those workers who extended their rotas – highlights that care recipients’ interests prevailed over the ones of live-in carers. This became further evident when care workers, as international commuters, were required to test or undergo quarantine while care recipients and their families were not subjected to similar requirements. Although live-in care is a transnational arrangement, the implementation of national and regional measures highlighted the relevance of national interests in the context of maintaining the live-in care model, while the interests of care workers remained secondary.

In March 2020, the European Commission published a series of guidelines for protecting public health while ensuring ‘the availability of goods and essential services’ as well as ‘the free movement of workers during COVID-19 outbreak’ (European Commission, 2020). According to EU guidelines, member states

should permit and facilitate the crossing of frontier workers, in particular but not only those working in the health care and food sector, and other essential services (e.g. child care, elderly care, critical staff for utilities) to ensure continued professional activity. (Official Journal of the European Union, C 102 I/03)

While these types of recommendations highlighted the fact that care workers are critical workers and contributed to their recognition in the public and at the policy level, the COVID-19 pandemic continues to exacerbate the economic pressure on care workers to accept precarious working conditions. At the same time, the pandemic has revealed that live-in care workers are in high demand, which could potentially increase their bargaining power (see Safuta and Noack, 2020). Protests of care workers at the Slovakian–Austrian border and care workers’ initiatives that received mainstream media coverage indicate that care workers’ voices will increasingly have to be taken into account by brokering agencies and policymakers alike.

## Conclusion

As from June 2020, live-in carers from Slovakia and Romania have slowly returned – although under strict anti-pandemic measures – to regular rotas. New travel restrictions, changing regulations regarding testing and quarantine, effective since the fall 2020, and controversies over the question whether live-in carers belong to the group of commuters who are exempted from border regulations (e.g. BMSGPK, Federal Ministry for Social Affairs, Health, Care and Consumer Protection, 2020a), however, show the unpredictability and uncertainty that live-in carers continue to be exposed to. As was shown, care workers’ interests have not been effectively addressed during the pandemic thus far, but instead subordinated to those of care recipients and their families, brokering agencies and sending and receiving countries. While workers with their limited bargaining power were left at the mercy of more powerful actors even before the pandemic, the national focus of pandemic-related responses drew renewed attention to the power imbalances as well as to the deeper structural inequalities, dependencies and forms of exploitation within Europe that underlie care mobility. Although the pandemic also underscored that migrant care workers are essential for health and long-term care systems, it remains to be seen whether the live-in care model (and the care workers’ position within) will evolve or remain unchanged. A pandemic-related worsening of the economic situation in the countries of origin could increase the financial pressure on care workers and thus their dependence on the job as a live-in. Therefore, a critical examination of the systems’ sustainability is imperative – including concerning transnational care mobility and its commuting workers.

## Notes

1.
https://www.facebook.com/curafair/

2.
https://www.facebook.com/dreptpentruingrijire/

3.
https://www.facebook.com/groups/241430186209702/

4. Further in-depth interviews with carers are required to substantiate the findings concerning their perspectives.

5. Correspondence with the Austrian Economic Chamber, statistics department, on 23 April 2020.

6. In Austria, self-employed have to pay into the public social insurance. Their coverage is similar, albeit less comprehensive compared to employees. Self-employed are not insured against unemployment, although this protection as well as, for example, sickness pay can be purchased by supplementary insurances.

7. This precondition was waived on 23 March 2020 for the duration of the pandemic.

8. Correspondence with the Austrian Economic Chamber, statistics department, on 23 April 2020.

9. In February 2020, almost 470,000 people received the public Long-Term Care Allowance (Statistik Austria, 2020), meaning that live-in care recipients accounted for 7% of all people with ascertained care needs. Austria relies heavily on familial care and, with 95,000 older adults in long-term care facilities (BMASGK, Federal Ministry of Labour, Social Affairs, Health and Consumer Protection, 2019), the 33,000 live-in care recipients make up a fourth of people with care needs that warrant an (almost) around-the-clock availability of care workers.

10. Care recipients were encouraged by some agencies to use the money they had saved as a result of lower travel expenses (as carers worked longer rotas) to pay for the tests.

11. Austrian citizens or people with residence in Austria were allowed to enter Austria if they went into 14-day self-isolation at home after mid-April. As personal carers have their residence registered with the older adult they care for, they had to quarantine in a hotel instead.

12. Romanian media reported the lack of physical distancing and, in general, a concern for workers’ health also when seasonal agricultural workers from Romania flew to Germany.

13. The province of Upper Austria had initially announced a bonus of 1000 euros. In practice, some care workers reportedly did not even receive the whole amount of 500 euros.

14. Correspondence with the Austrian Economic Chamber, statistics department, on 23 April 2020.

15. Even though required to keep all receipts, if earnings are below the tax threshold, no self-employee is required to submit for annual income tax assessment at the tax office.

16. In January 2019, the European Commission opened infringement proceedings against Austria because of the family allowance adjustment, which the Commission deems a treaty violation. As no political resolution was achieved, the Commission brought a complaint before the European Court of Justice in May 2020.

17. Both live-in care workers and agencies are (formally) represented by the Economic Chamber. Interviews revealed that, when contacted by care workers, chamber representatives refuse to mediate between care workers and agencies as both are members.

## References

[bibr1-14680181211008340] Anderson B (2000) Doing the Dirty Work? The Global Politics of Domestic Labour. London: Zed Books.

[bibr2-14680181211008340] Anderson B Shutes I (eds) (2014) Migration and Care Labour: Theory, Policy and Politics. Basingstoke: Palgrave Macmillan.

[bibr3-14680181211008340] Aulenbacher B Leiblfinger M Prieler V (2020) The promise of decent care and the problem of poor working conditions: Double movements around live-in care in Austria. Socialpolicy.ch – Journal of the Division of Sociology, Social Policy, Social Work 2: 2.5.

[bibr4-14680181211008340] Aulenbacher B Leiblfinger M Prieler V (2021) Das umstrittene Selbstständigenmodell: Live-in-Betreuung in Österreich. In: Aulenbacher B Lutz H Schwiter K (eds) Gute Sorge ohne gute Arbeit? Live-in-Care in Deutschland, Österreich und der Schweiz. Weinheim; Basel: Beltz Juventa, pp. 66–78.

[bibr5-14680181211008340] Bahna M (2014) Slovak care workers in Austria: How important is the context of the sending country? Journal of Contemporary European Studies 22(4): 411–426.

[bibr6-14680181211008340] Bahna M Sekulová M (2019) Crossborder Care: Lessons from Central Europe. Cham: Palgrave Macmillan.

[bibr7-14680181211008340] Bauer G Österle A (2013) Migrant care labour: The commodification and redistribution of care and emotional work. Social Policy and Society 12(3): 461–473.

[bibr8-14680181211008340] BMASGK, Federal Ministry of Labour, Social Affairs, Health and Consumer Protection (2019) Österreichischer Pflegevorsorgebericht 2018. Available at: https://broschuerenservice.sozialministerium.at/Home/Download?publicationId=719 (accessed 6 October 2020).

[bibr9-14680181211008340] BMF, Federal Ministry of Finance (2020) Härtefallfonds: 6 statt 3 Zahlungen, bis zu 3.000 Euro Comeback-Bonus. Available at: https://www.bmf.gv.at/presse/pressemeldungen/2020/Mai/haertefallfonds.html (accessed 30 July 2020).

[bibr10-14680181211008340] BMSGPK, Federal Ministry for Social Affairs, Health, Care and Consumer Protection (2020a) 24-Stunden-Betreuung. Änderung der Verordnung über die Einreise nach Österreich im Zusammenhang mit der Eindämmung von SARS-CoV-2 im Zusammenhang mit 24-Stunden-Betreuungskräften, Geschäftszahl: 2020-0.537.480, 24 August. Available at: https://www.wko.at/branchen/w/gewerbe-handwerk/personenberatung-betreuung/personenbetreuung/2020-08-24-Aenderung-VO-Einreise-24-Stunden-Betreuung.pdf (accessed 15 September 2020).

[bibr11-14680181211008340] BMSGPK, Federal Ministry for Social Affairs, Health, Care and Consumer Protection (2020b) ANSCHOBER: Bundesregierung arbeitet mit Hochdruck an Lösung für 24h BetreuerInnen. Press release. Available at: https://www.ots.at/presseaussendung/OTS_20200314_OTS0040 (accessed 9 September 2020).

[bibr12-14680181211008340] Bratislavske Noviny (2020) Minister vnútra reaguje na včerajší protest opatrovateliek na hraničnom priechode Petržalka–Berg, 27 April. Available at: https://www.bratislavskenoviny.sk/policia/59596-minister-vnutra-reaguje-na-vcerajsi-protest-opatrovateliek-na-hranicnom-priechode-petrzalka-berg (accessed 16 September 2020).

[bibr13-14680181211008340] Casanova G Di Rosa M Fisher O , et al. (2020) Between migrant care work and new occupational welfare tools: Changing home care arrangements in Italy. International Journal of Environmental Research and Public Health 17(15): 5511. 10.3390/ijerph17155511 PMC7432092 32751605

[bibr14-14680181211008340] CFR Călători (2020) Transport special în Austria, 16 May. Available at: https://www.cfrcalatori.ro/comunicate/transport-special-in-austria/ (accessed 22 May 2020).

[bibr15-14680181211008340] Chau HS (2020) Brokering Circular Labour Migration: A Mobile Ethnography of Migrant Care Workers’ Journey to Switzerland. Abingdon: Routledge.

[bibr16-14680181211008340] Cox R (2006) The Servant Problem: Domestic Employment in a Global Economy. London: I. B. Tauris.

[bibr17-14680181211008340] European Commission (2020) Proposal for a Council Recommendation on a coordinated approach to the restriction of free movement in response to the COVID-19 pandemic. COM(2020) 499 final. Available at: https://ec.europa.eu/info/sites/info/files/council-proposal-coordinated-approach-restriction-movement_en.pdf (accessed 8 October 2020).

[bibr18-14680181211008340] Giordano C (2021) Freedom or money? The dilemma of migrant live-in elderly carers in times of COVID-19. Gender, Work & Organization 28(S1): 137–150. 10.1111/gwao.12509 PMC7361578 32837020

[bibr19-14680181211008340] Habel S Tschenker T (2020) Stay at work: Zur Situation der Live-In-Pflege in der Corona-Krise. Soziale Sicherheit 69(6): 215–219.

[bibr20-14680181211008340] Haidinger B (2016) Flexibilität, Absicherung und Interessenvertretung in der 24-Stunden-Betreuung . . . grenzenlos? In: Weicht B Österle A (eds) Im Ausland zu Hause pflegen: Die Beschäftigung von MigrantInnen in der 24-Stunden-Betreuung. Wien: LIT, pp. 87–114.

[bibr21-14680181211008340] Hochschild AR (2001) Global care chains and emotional surplus value. In: Hutton W Giddens A (eds) On the Edge: Living with Global Capitalism. London: Vintage, pp. 130–146.

[bibr22-14680181211008340] Horn V Schweppe C (2020) Häusliche Altenpflege in Zeiten von Corona: Erste Studienergebnisse. Report, Johannes Gutenberg-Universität Mainz, Mainz.

[bibr23-14680181211008340] Horn V Schweppe C Böcker A , et al. (2019) Live-in migrant care worker arrangements in Germany and the Netherlands: Motivations and justifications in family decision-making. International Journal of Ageing and Later Life 13(2): 83–113.

[bibr24-14680181211008340] Kofman E Raghuram P (2015) Gendered Migrations and Global Social Reproduction. Basingstoke: Palgrave Macmillan.

[bibr25-14680181211008340] Koštialiková K (2020) Opatrovateľkám dochádzajú psychické aj fyzické sily. Štátna karanténa je pre nich nočnou morou. Zoznam.sk, 24 April. Available at: https://glob.zoznam.sk/rozhovor-opatrovatelkam-dochadzaju-psychicke-aj-fyzicke-sily-statna-karantena-je-pre-nich-nocnou-morou/ (accessed 16 September 2020).

[bibr26-14680181211008340] Leiber S Rossow V Österle A , et al. (2020) Yet another black box: Brokering agencies in the evolving market for live-in migrant care work in Austria and Germany. International Journal of Care and Caring. Epub ahead of print 24 November 2020. DOI: 10.1332/239788220X15988973352874.

[bibr27-14680181211008340] Leiblfinger M Prieler V (2020) Updates on Migrant Live-in Care in Austria at the Time of COVID-19: A Glimpse into the Media. ILPN’s LTCCovid. Available at: https://ltccovid.org/2020/04/10/updates-on-migrant-live-in-care-in-austria-at-the-time-of-covid-19-a-glimpse-into-the-media1/ (accessed 24 June 2020).

[bibr28-14680181211008340] Leiblfinger M Prieler V Schwiter K , et al. (2020) Impact of COVID-19 policy responses on live-in care workers in Austria, Germany, and Switzerland. Journal of Long-Term Care 2020: 144–150.

[bibr29-14680181211008340] Leichsenring K Schmidt AE Bauer A (2020a) Report: Planning for Expected Shortages in Migrant and Family Care in Austria. ILPN’s LTCCovid. Available at: https://ltccovid.org/2020/03/26/report-planning-for-expected-shortages-in-migrant-and-family-care-in-austria/ (accessed 30 March 2020).

[bibr30-14680181211008340] Leichsenring K Staflinger H Bauer A (2020b) The Situation of ‘24-Hour Care’ from the Perspective of Migrant Caregivers in Austria. ILPN’s LTCCovid. Available at: https://ltccovid.org/2020/04/08/the-situation-of-24-hour-care-from-the-perspective-of-migrant-caregivers-in-austria/ (accessed 6 May 2020).

[bibr31-14680181211008340] Lupu G (2020) Îngrijitoarele românce din Austria: “Vrem să muncim indiferent de condiții”. Umilințele la care sunt supuse în timpul pandemiei. B1, 1 April. Available at: https://www.b1.ro/stiri/eveniment/ingrijitoarele-romance-in-austria-vrem-sa-muncim-indiferent-de-conditii-conditiile-precare-la-care-sunt-supuse-in-timpul-pandemiei-327413.html (accessed 20 September 2020).

[bibr32-14680181211008340] Lutz H (2018) Care migration: The connectivity between care chains, care circulation and transnational social inequality. Current Sociology 66(4): 577–589.

[bibr33-14680181211008340] Lutz H (2020) 24-Stunden-Pflege im Sperrmodus: Care-Migration in Zeiten von Covid 19. Covid 19 – Center Blog des CGC. Available at: http://www.cgc.uni-frankfurt.de/176487/24-stunden-pflege-im-sperrmodus-care-migration-in-zeiten-von-covid-19/ (accessed 10 September 2020).

[bibr34-14680181211008340] Matei F (2020) Wie sich 24-Stunden-Betreuer*innen in der Corona-Krise organisieren. In: mosaik – politik neu zusammensetzen. Available at: https://mosaik-blog.at/wie-sich-24-stunden-betreuerinnen-in-der-corona-krise-organisieren/ (accessed 9 September 2020).

[bibr35-14680181211008340] Matzal A (2020) Izolați în lumea largă. „Noi suntem cei pierduți pe drumuri”. Scena9, 25 March. Available at: https://www.scena9.ro/article/diaspora-coronavirus-pandemie-ingrijitori-austria (accessed 20 September 2020).

[bibr36-14680181211008340] Melegh A Gábriel D Gresits G , et al. (2018) Abandoned Hungarian workers and the political economy of care work in Austria. Szociológiai Szemle (Review of Sociology) 28(4): 61–87.

[bibr37-14680181211008340] Meseșan D (2020) Interesele din spatele zborurilor private care duc sute de îngrijitoare din România la muncă în Austria, în plină pandemie. Oficial, sunt „repatriate”. Libertatea, 8 April. Available at: https://www.libertatea.ro/stiri/interesele-din-spatele-zborurilor-private-care-duc-sute-de-ingrijitoare-din-romania-la-munca-in-austria-in-plina-pandemie-oficial-sunt-repatriate-2944335 (accessed 20 September 2020).

[bibr38-14680181211008340] Miedl M (2020) 24-Stunden-Pflege: Auf die Füße stellen, laut werden, solidarisch sein. A&W Online, 10 August. Available at: https://www.arbeit-wirtschaft.at/reportage-24-stunden-pflege/ (accessed 24 August 2020).

[bibr39-14680181211008340] Ministerul Transporturilor, Infrastructurii și Comunicațiilor (2020) Precizări, 1 May. Available at: http://www.mt.gov.ro/web14/spatiul-media/comunicate-de-presa/2928-01052020 (accessed 20 September 2020).

[bibr40-14680181211008340] Official Journal of the European Union (2020) Communication from the Commission: Guidelines Concerning the Exercise of the Free Movement of Workers during COVID-19 Outbreak. 2020/C 102 I/03. Available at: https://eur-lex.europa.eu/legal-content/EN/TXT/?uri=CELEX%3A52020XC0330%2803%29 (accessed 08 October 2020).

[bibr41-14680181211008340] Österle A (2016) 24-Stunden-Betreuung und die Transnationalisierung von Pflege und Betreuung: Aktuelle Dimensionen und wohlfahrtsstaatliche Implikationen. In: Weicht B Österle A (eds) Im Ausland zu Hause pflegen: Die Beschäftigung von MigrantInnen in der 24-Stunden-Betreuung. Wien: LIT, pp. 247–269.

[bibr42-14680181211008340] Österle A Bauer G (2016) The legalization of rotational 24-hour care work in Austria: Implications for migrant care workers. Social Politics: International Studies in Gender, State & Society 23(2): 192–213.

[bibr43-14680181211008340] Österle A Hasl A Bauer G (2013) Vermittlungsagenturen in der 24-h-Betreuung. WISO – Wirtschafts- und Sozialpolitische Zeitschrift 36(1): 159–172.

[bibr44-14680181211008340] Parreñas RS (2015) Servants of Globalization: Migration and Domestic Work, 2nd edn. Stanford, CA: Stanford University Press.

[bibr45-14680181211008340] Pelzelmayer K (2016) Places of difference: Narratives of heart-felt warmth, ethnicisation, and female care-migrants in Swiss live-in care. Gender, Place & Culture 23(12): 1701–1712.

[bibr46-14680181211008340] Petrovič J Debnár J (2020) Koronavírus: Opatrovateľky protestovali na priechode Berg, aby im uznali zahraničné testy. Aktuality, 26 April. Available at: https://www.aktuality.sk/clanok/785681/koronavirus-opatrovatelky-protestuju-na-priechode-berg-aby-im-uznali-zahranicne-testy/ (accessed 16 September 2020).

[bibr47-14680181211008340] Safuta A Noack K (2020) A pandemic, and then what? The effects of the coronavirus pandemic on migrant care workers in Germany. Routed Magazine 10. Available at: https://www.routedmagazine.com/care-workers-germany (accessed 29 June 2020).

[bibr48-14680181211008340] Schilliger S Schwiter K Steiner J , et al. (2020) Grenzerfahrungen in der Betagtenbetreuung. WOZ Die Wochenzeitung, 7 May. Available at: https://www.woz.ch/2019/care-arbeit/grenzerfahrungen-in-der-betreuung-von-betagten? (accessed 11 May 2020).

[bibr49-14680181211008340] Schmidt AE Winkelmann J Rodrigues R , et al. (2016) Lessons for regulating informal markets and implications for quality assurance – The case of migrant care workers in Austria. Ageing & Society 36(4): 741–763.

[bibr50-14680181211008340] Schwiter K Steiner J (2020) Geographies of care work: The commodification of care, digital care futures and alternative caring visions. Geography Compass 14(12): e12546.

[bibr51-14680181211008340] Sekulová M (2013) Transnational households in the context of female migration from Slovakia to Austria. Lidé města / Urban People 15(2): 217–236.

[bibr52-14680181211008340] Sekulová M (2020) Cezhraničná cirkulácia starostlivosti a chýbajúci hlas opatrovateliek. Kapitál, 7 May. Available at: https://kapital-noviny.sk/cezhranicna-cirkulacia-starostlivosti-a-chybajuci-hlas-opatrovateliek/ (accessed 12 October 2020).

[bibr53-14680181211008340] Sekulová M Rogoz M (2019) The perceived impacts of care mobility on sending countries and institutional responses: Healthcare, long-term care and education in Romania and Slovakia. REMINDER working paper. Available at: https://www.reminder-project.eu/wp-content/uploads/2019/01/REMINDER-D6.2-Perceived-Impacts-of-Care-Work-Mobility.pdf (accessed 12 October 2020).

[bibr54-14680181211008340] Statistik Austria (2020) Bundespflegegeldbezieherinnen und -bezieher sowie Ausgaben für das Bundespflegegeld 2020. Available at: http://www.statistik.at/web_de/statistiken/menschen_und_gesellschaft/soziales/sozialleistungen_auf_bundesebene/bundespflegegeld/index.html (accessed 06 October 2020).

[bibr55-14680181211008340] Steiner J Prieler V Leiblfinger M , et al. (2019) Völlig legal!? Rechtliche Rahmung und Legalitätsnarrative in der 24h-Betreuung in Deutschland, Österreich und der Schweiz. Österreichische Zeitschrift für Soziologie 44(1): 1–19.

[bibr56-14680181211008340] Uhde Z (2016) From women’s struggles to distorted emancipation. International Feminist Journal of Politics 18(3): 390–408.

[bibr57-14680181211008340] Van Hooren F (2020) Covid-19, migrant workers and the resilience of social care in Europe. European University Institute. Available at: https://migrationpolicycentre.eu/docs/migreshub/MigResHub-think-piece-No4.pdf (accessed 1 March 2021).

[bibr58-14680181211008340] Vojenčák R (2020) Každá opatrovateľka, ktorá uviazla v zahraničí má právo sa rozhodnúť, či počká tam, alebo sa vráti’. SME, 19 March. Available at: https://mynovohrad.sme.sk/c/22362753/kazda-opatrovatelka-ktora-uviazla-v-zahranici-ma-pravo-sa-rozhodnut-ci-pocka-tam-alebo-sa-vrati.html (accessed 16 September 2020).

[bibr59-14680181211008340] Weicht B (2015) Employment without employers? The public discourse on care during the regularisation reform in Austria. In: Triandafyllidou A Marchetti S (eds) Employers, Agencies and Immigration: Paying for Care. Farnham; Burlington, VT: Ashgate, pp. 113–130.

[bibr60-14680181211008340] Weicht B Österle A (eds) (2016) Im Ausland zu Hause pflegen: Die Beschäftigung von MigrantInnen in der 24-Stunden-Betreuung. Wien: LIT.

[bibr61-14680181211008340] Williams F (2011) Towards a transnational analysis of the political economy of care. In: Mahon R Robinson F (eds) Feminist Ethics and Social Policy: Towards a New Global Political Economy of Care. Vancouver, BC, Canada; Toronto, ON, Canada: University of British Columbia Press, pp. 21–38.

[bibr62-14680181211008340] Williams F (2018) Care: Intersections of scales, inequalities and crises. Current Sociology 66(4): 547–561.

[bibr63-14680181211008340] Winkelmann J Schmidt AE Leichsenring K (2015) Regulating migrants as a low-cost solution for long-term care: The formalisation of a dual care labour market in Austria. In: Carbonnier C Morel N (eds) The Political Economy of Household Services in Europe. New York: Palgrave Macmillan, pp. 172–194.

[bibr64-14680181211008340] WKO, Economic Chamber Austria, Statistics Department (2020) Personenberatung und Personenbetreuung: Branchendaten. Available at: http://wko.at/statistik/BranchenFV/B_127.pdf (accessed 9 May 2020).

